# Age and phenology control photosynthesis and leaf traits in the understory woody species, *Rhamnus cathartica* and *Prunus serotina*

**DOI:** 10.1093/aobpla/plac044

**Published:** 2022-09-30

**Authors:** Mary Heskel, Jean Pengra, Ally Kruper, Michael D Anderson, Jerald J Dosch, Lianna Goldstein, Shannon Hahn, Sarah Hoffman

**Affiliations:** Department of Biology, Macalester College, Saint Paul, MN 55105, USA; Department of Biology, Macalester College, Saint Paul, MN 55105, USA; Department of Biology, Macalester College, Saint Paul, MN 55105, USA; Department of Biology, Macalester College, Saint Paul, MN 55105, USA; Department of Biology, Macalester College, Saint Paul, MN 55105, USA; Department of Biology, Macalester College, Saint Paul, MN 55105, USA; Department of Biology, Macalester College, Saint Paul, MN 55105, USA; Department of Biology, Macalester College, Saint Paul, MN 55105, USA

**Keywords:** Fluorescence, invasive species, non-native species, photosynthesis, seasonality, stomata, temperate forest, understory

## Abstract

Understory plants are often inadequately represented or neglected within analyses of forest ecosystem productivity. Further, the potential impacts of the biological factors of age class and growth form on carbon cycling physiology, and how it may vary across the growing season and amongst species of different native/non-native status, have not been thoroughly considered. Our study examines photosynthesis and associated physical leaf traits in two understory woody species, *Rhamnus cathartica*, introduced and invasive in North America, and *Prunus serotina*, a common subcanopy species native to North America. We estimated leaf-level photosynthesis as measured through light and carbon dioxide response curves, dark-adapted chlorophyll fluorescence and leaf traits (leaf mass per area and stomatal density) for each combination of species and age class at plots in the understory of a temperate deciduous research forest in the US Upper Midwest at two time points during the growing season, late spring (late May) and mid-summer (mid-July). Carbon assimilation rates from light response curves (*A*_sat_, *A*_400_) and fluorescence capacity estimate *F*_v_/*F*_m_ all increased between the two measurement points in both species and age class. Estimates of carbon reaction capacity (*V*_cmax_ and *J*_max_) exhibited a different directional response to seasonal development, declining in seedlings of both species and *P. serotina* trees (~8–37 % reduction in *V*_cmax_, ~9–34 % reduction in *J*_max_), though increased in trees of *R. cathartica* (+24 % in *V*_cmax_, +9 % in *J*_max_). Divergent responses in photosynthetic parameters amongst these factors may be explained by species differences in leaf mass per area and stomatal density, which together are likely influenced by both growth form, canopy position and ontogeny. Overall, we believe our findings suggest complex, varied influences on photosynthesis that indicate environmental and biological plasticity which may contribute to the historic and continued expansion of *R. cathartica* in the US Upper Midwest region.

## Teaser

Shaded, understory leaves are often under-represented when considering forest productivity. Our study closely examines how two common understory woody species vary in photosynthesis and leaf traits through the growing season in a temperate forest, and how this relates to their age class and status as native and non-native, invasive species. We found varied responses that overall suggest competitive carbon gain and light use strategies of the invasive shrub, *Rhamnus cathartica*, which may help to explain its sustained dominance in the region.

## Introduction

Shade plants are often under-represented or specifically excluded from global databases of plant traits that are used for models of ecosystem productivity (Niinemets *et al.* 2015; Keenan and Niinemets 2016). However, small trees, seedlings and saplings, in addition to understory shrubs and forbs, provide a large proportion of the carbon assimilation capacity that must be accounted to fully characterize gross and net primary productivity of a forested area (He *et al.* 2018). As shaded leaves comprise a substantial contribution to forest productivity, but are buffered from light and temperature extremes due to their canopy position, they are poised to be increasingly important in maintaining carbon cycling as climate change pushes air temperatures beyond historic norms (He *et al.* 2018). Diffuse light experienced by understory seedlings and trees may provide growth environments that optimize photosynthetic uptake by decreasing photoinhibition (Williams *et al.* 2014), though the more limited light environment may likely reduce photosynthesis and growth rates compared to individuals growing in gaps and full light (Brodersen *et al.* 2008; Zhang *et al.* 2013). Where species fall along the shade tolerance–intolerance spectrum can determine their growth and photosynthetic response, as observed in controlled experiments (Kothari *et al.* 2021).

In boreal forests and other forest ecosystems with lower canopy leaf area index values, understory vegetation may drive ecosystem carbon cycling (Nilsson and Wardle 2005; Kolari *et al.* 2006; Ikawa *et al.* 2015). In temperate deciduous forests, where canopy leaf area index is generally higher than boreal forests and more completely shades the understory environment, understory vegetation can adapt to maximize seasonal photosynthetic gain early in spring prior to canopy closure, and later in autumn after senescence (Harrington *et al.* 1989; Wei *et al.* 2017; Heberling *et al.* 2019). While these adaptations suggest a seasonal plasticity of physiological capacity driven by light and development, it is less clear how understory trees vary amongst age classes—from young, shaded seedlings to mature, yet still understory, adult trees, within and across species.

Previous research comparing understory seedlings to adult canopy trees provided insights on the combined controls of ontology and environment (Wilson *et al.* 2000). A broad meta-analysis on physiological rates and associated leaf traits of saplings and adult trees found higher rates of area-based photosynthesis as well as leaf mass per area (LMA) in adult canopy trees compared to younger individuals of the same species (Thomas and Winner 2002). Higher photosynthetic rates in mature trees may be due to increased leaf nitrogen compared to seedlings (Mediavilla and Escudero 2003), or a greater ability to acclimate to high-light environments (Cai *et al.* 2005). In forest trees, age differences in terms of area-based carbon assimilation are most pronounced in temperate deciduous species, compared to tropical evergreen and conifers (Thomas and Winner 2002), suggesting that plant functional type and climate may alter the interaction of physiology and age. We apply this groundwork of understanding of age-based variability in forest carbon cycling in our study to further examine these drivers across the growing season and different species.

Differences in accumulated growth temperatures between the microenvironments of saplings and adult canopy trees may drive changes in physiology and phenology within the same tree species. Earlier budbreak and similarly timed senescence in understory saplings compared to canopy trees provide longer periods of potential carbon assimilation (Augspurger and Bartlett 2003). However, not all deciduous conspecific understory and canopy trees vary their budbreak similarly, complicating how to interpret understory adaptations of forest species (Richardson and O’Keefe 2009). Earlier leaf emergence offers the advantage of pre-canopy-closure light, but also the potential risk of frost damage in seasonally cold climates (Richardson and O’Keefe 2009; Laube *et al.* 2014). It is unclear if leaves of mature understory trees act similarly to leaves of immature upper-canopy saplings, even if they occupy a similar micro-environment within the forest, either due to plasticity in their development or species-specific variability in carbon cycling responses. Understory tree saplings can adjust both timing and capacity of leaf processes to make use of brief light and temperature advantages, so it is reasonable to ask—do understory trees and their conspecific seedlings, which have a narrower range of microclimate differences, respond similarly?

A further nuance to consider when comparing how understory trees occupy and use resources across age class is their native/non-native status in a region. In the North American Midwest, *Rhamnus cathartica* (common buckthorn), an introduced species that is endemic in Asia and Europe, continues to occupy and expand in temperate forest understories and edges, in many cases, potentially outcompeting native species that occupy similar niches, such as *Prunus serotina* (black cherry), for above- and below-ground resources (Knight *et al.* 2007). The physiology of *R. cathartica* may provide insight into its ability to expand in the understory: while it can tolerate shade, it effectively can also take advantage of sun availability more rapidly than other species grown under similar conditions (Grubb *et al.* 1996) and exhibits high values of leaf nitrogen and rates of photosynthesis in the understory (Harrington *et al.* 1989; Knight *et al.* 2007). By comparison, *P. serotina* is endemic to North America, but introduced and aggressively invasive in Europe (Godefroid *et al.* 2005; Closset-Kopp *et al.* 2011). In its native range, *P. serotina* is characterized by its large presence in oak forest understories and varies in its shade and drought tolerance (Auclair and Cottam 1971; Abrams *et al.* 1992). *Prunus serotina* is also not known for extended leaf phenology adaptation, which is widely observed in *R. cathartica* in North America (Knight *et al.* 2007). By examining carbon cycling and leaf traits of *R. cathartica* and *P. serotina* individuals of different age classes and different understory microenvironments, we can begin to understand how these species vary in their ability to grow and expand in temperate forests. Comparing native and non-native species in the same understory limits the influence of many potentially confounding variables to achieve a relatively representative characterization of how these species assimilate carbon and grow across age classes.

Understory trees in temperate deciduous forests occupy a functional niche that requires examination in terms of their role in the ecosystem carbon cycle. It remains unclear how low- and mid-canopy conspecifics that represent different age classes vary in their ability to assimilate carbon and grow in a shaded environment. As prior studies have primarily focused on shaded versus unshaded or seedling versus adult comparisons of canopy trees, we lack the necessary data to characterize how understory trees function in forest field conditions (Harrington *et al.* 1989; Cai *et al.* 2005; Knight *et al.* 2007; Brodersen *et al.* 2008; Heberling *et al.* 2019). As many non-native and invasive species in North America are understory, and not canopy, species, understanding how they function may be crucial to forecasting future forest biodiversity and carbon balances (Xu *et al.* 2007a; Heberling *et al.* 2019). To address these concerns, we examine photosynthetic and leaf traits in adults and seedlings of two understory tree species that are common to the US Midwest: *P. serotina* and *R. cathartica*. We hypothesize that *R. cathartica*, a highly competitive invasive species in the study region, will optimize photosynthesis at both seedling and adult age classes compared with *P. serotina*, contributing towards its competitive growth and expansion in low- and mid-canopy forest levels. We also hypothesize photosynthetic optimization earlier in the growing season for *R. cathartica*, which would align with its observed earlier leaf phenology, compared to *P. serotina*.

## Materials and Methods

### Field site and study species

All sampling occurred at Katharine Ordway Natural History Study Area (‘Ordway’), a field station located in Inver Grove Heights, Dakota County, MN, USA, operated by Macalester College (Saint Paul, MN, USA). Both Ordway and Macalester’s Saint Paul campus reside on the ancestral and contemporary lands of the Sisseton and Wahpeton bands of the Dakota people. Ordway’s landscape consists of tallgrass prairie, freshwater wetland and deciduous forest ecosystems, and details of these ecosystems are described in Davis *et al.* (2012). The deciduous forest system is dominated by oak species, including *Quercus rubra* and *Quercus alba*, and contains a biodiversity reflective of other deciduous forests in the Upper North American Midwest region. Recent windfall events have altered the canopy composition, and allowed for higher light availability in parts of the understory (Anderson *et al.* 2019), though we aimed to avoid said light gaps for site selection in this study. *In situ* environmental and physiological measurements took place in the understory of Ordway’s forest to capture a representative perspective on the non-canopy shrubs/low-stature trees, *R. cathartica* and *P. serotina*.

Our experimental design aimed to collect a representative sampling of seedlings and trees of *R. cathartica* and *P. serotina* in the Ordway understory. To do this, we selected four sites, of roughly a 5-m radius within the forest a minimum of 12 m apart from each other (maximum distance between sites ~100 m), with no clear sign of windfall disturbance or light gaps, where a seedling and tree of each species were present. These sites of paired species and age classes were considered an experimental block. This design allowed us to compare across age classes and species at the same site, given near-identical environmental variables in a forest with diverse terrain and light availability. We acknowledge there is a non-zero likelihood of seedlings originating from the nearby trees of the same species, creating a parent–offspring genetic relationship that might impact physiological and morphological traits. Testing the relatedness of the species within and across blocks and how this might impact our variables of interests lies beyond the scope of this analysis; however, we address this potential influence and the variation it might add by using a mixed model that included a random effect of ‘site’. Seedlings of both species were defined as individuals smaller than 1 m in height, with minimal branching, diameter at breast height (DBH) of less than 2.5 cm, and likely representing an age less than 5 years old given the known growth patterns of both species. Individuals that we classified as trees had to meet the following criteria: taller than 2 m and DBH > 4 cm. The values for DBH at our sites for trees ranged from 7.0 to 12.6 cm, and leaves were sampled from the canopy at ~1.25–1.75 m in height. At Ordway, the population structure of these species is such that there were many seedlings, and few trees, at each site. In this study, we aimed to capture differences during the growing season that understory trees and seedlings experience at two time points during the growing season. Sampling occurred during the growing season of 2019, with 2-week-long field campaigns: one in late May extending through early June (‘Early season’), and the other in mid-July (‘Mid season’). Budburst at Ordway generally occurs in mid-May, with deciduous leaf expansion continuing through mid-June.

### Leaf-level gas exchange

At each experimental site (*n* = 4), one fully expanded leaf from a seedling and tree of each species was selected for gas exchange measurements, which included a light response curve and a CO_2_ response curve in succession. Leaves chosen for gas exchange measurements were often the furthest from a branching point in both trees and seedlings to enable greater maneuverability with the equipment and tripod that supported the cuvette; our study did not have the resources to employ a bucket-lift, so all branches were reachable from the ground, at heights of ~1.25–1.75 m in height. To do this, we used an infrared gas analyser (IRGA; Li-COR 6800, Li-COR Biosciences, Lincoln, NE, USA) with a 3 × 3 cm leaf cuvette. Leaves were placed inside the cuvette to maximize leaf area measured. For the light response curves, we set reference CO_2_ concentration to 410 ppm, to represent the current global atmospheric CO_2_ concentration in 2019 during the Northern Hemisphere summer. Cuvette relative humidity levels were maintained at 50 % to enhance photosynthesis measurements, and replicate field conditions in the understory. Cuvette temperature was allowed to reflect ambient conditions of the understory, which ranged from 18–27 °C amongst the Early and Mid season sampling campaigns. Based on relative humidity and ambient temperature ranges, the vapour pressure deficit of the inside the cuvette chamber ranged from 1.04 to 1.81 kPa. Prior to the light response curves, leaves in the cuvette acclimated to measurement conditions for a minimum of 5 min under a light level of 400 μmol m^−2^ s^−1^ photosynthetically active radiation (PAR), the first measurement point of the light curve, and an intensity representative of the understory environment. The light response curves were comprised of 13 light levels: 400, 800, 1000, 1200, 800, 400, 300, 200, 100, 50, 20, 10 and 0 μmol m^−2^ s^−1^ PAR, with leaves experiencing a minimum of 90 s and a maximum of 120 s at each light level to achieve stability in CO_2_ and water measurements prior to logging a data point.

After light response curves were completed, we set the internal cuvette conditions to re-acclimate the leaf prior to inducing a CO_2_ response curve (*A*–*C*_i_ curve): CO_2_ reference was set to 400 ppm, and PAR to 400 μmol m^−2^ s^−1^, with relative humidity and flow rate maintained at the same levels in the light response curve. We allowed leaves to acclimate to pre-*A*–C_*i*_ curve conditions for a minimum of 5 min, or until stability in CO_2_ and water fluxes were achieved. For *A*–C_*i*_ curves, PAR was maintained at 400 μmol m^−2^ s^−1^ for the entirety of the measurement, to represent ambient understory light. The 12 *A*–C_*i*_ curve CO_2_ concentration levels were set at: 400, 300, 200, 100, 50, 0, 400, 400, 600, 800, 1000 and 1200 μmol m^−2^ s^−1^. As in the light response curves, a single leaf from each site × species × age class combination was measured, for a replicate number of four total curves. If leaves did not fully fill the 3 × 3 cm leaf chamber, we marked on the leaf the area present for measurement, and measured the exact area with a scanner (see below section) in order to area-correct the flux values.

After field-based physiological measurements, gas exchange data collected with the infrared gas analyzer (IRGA) were area-corrected and individual response curves were analysed for variables of interest. To analyse the light response curves, we used a function that fit our collected data to a non-rectangular hyperbola model, based on R code written by Nick Tomeo (Tomeo 2019). The parameters estimated from the light response curve include: light-saturated photosynthesis (*A*_sat_), mitochondrial dark respiration (*R*_d_) and photosynthesis at 400 μmol m^−2^ s^−1^ PAR (*A*_400_) to represent a more accurate understory light level. We also calculated a metric to estimate ‘carbon gain efficiency’ (CGE), which approximates the carbon gained per carbon exchanged at saturating light (CGE_sat_ = *A*_sat_/*A*_sat_ + *R*_d_) and a lower PAR value of 400 μmol m^−2^ s^−1^ (CGE = *A*_400_/*A*_400_ + *R*_d_). The CGE metric serves as an integrative view of carbon cycling at the leaf level, and can inform the broader processes.

Data from *A*–*C*_i_ curves were analysed using the ‘fitacis’ function in the R package ‘plantecophys’ (Duursma 2015). The data inputs for this package include values of estimated *C*_i_ (intercellular CO_2_ concentration), leaf temperature, PAR and calculated photosynthesis rates. The ‘fitaci’ function fits the Farquhar–Berry–von Caemmerer model of photosynthesis (Farquhar *et al.* 1980) to provide estimates of the following photosynthetic parameters in C_3_ plants: maximum rate of electron transport (*J*_max_) and maximum rate of RuBP (ribulose-1,5-bisphosphate carboxylation; *V*_cmax_).

### Chlorophyll fluorescence

To estimate the efficiency of photosystem II (PSII), *F*_v_/*F*_m_, in leaves of both species, we measured chlorophyll *a* fluorescence using a field-portable PAM fluorometer (Walz PAM Jr., Heinz Walz GmbH, Eiffeltrich, Germany) on dark-adapted leaves. Selected leaves (*n* = 4–5) from seedlings and trees of both species were fully covered in aluminium foil for a minimum of 20 min prior to measurement. To measure leaves, the fibre optic probe was fixed onto the adaxial side of the leaf, and held in place with magnets. *F*_v_/*F*_m_ can be calculated from the initial fluorescence emission from the leaf (*F*_0_), the maximal fluorescence value (*F*_m_) as follows: (*F*_m_ − *F*_0_)/*F*_m_, where *F*_m_ − *F*_0_ = *F*_v_. In the region’s ecosystems, Eriophyid mites (likely *Eriophyes cerasicrumena*) form finger-like galls on leaves of *P. serotina* (Garon-Labrecque 2017). A large number of *P. serotina* individuals are infected with *Eriophyes* spp., which form galls on the leaves, often in high density, likely complicating the leaf’s ability to capture and use light for photosynthesis. To compare the potential impact of gall formation on chlorophyll fluorescence, we measured *F*_v_/*F*_m_ in leaves of *P. serotina* (trees only, not seedlings) with and without galls present.

### Stomatal characteristics

At the time of the first physiological field measurements in late May and the beginning of June (Early season campaign), leaves not measured for photosynthesis measurements were destructively sampled to assess stomatal characteristics. To do this, we used a well-established, simple method (Grant and Vatnick 2004) that uses clear nail polish to create impressions of the leaf abaxial surface of 40 leaves of each species. Nail polish was applied in a length of ~1 cm to the leaf and left to dry for a minimum of 10 min. A length of clear plastic adhesive tape was then pressed on the dried polish on the leaf, and carefully peeled off to then be adhered to a glass microscope slide. The impression of the cells, including stomata, on the abaxial side of the leaf was then able to be viewed with a compound light microscope (Olympus CX21, Olympus Life Science, Tokyo, Japan) at ×40 magnification. For each impression peel collected from a leaf, three ‘field of views’ were assessed for the total number of stomata visible. From these counts, stomatal density (count per mm^2^) was calculated, based on the known area of the field of view of the microscope. A subsample of dried leaves from both species was imaged using a scanning electron microscope to visualize the abaxial leaf surface with higher resolution. Images of these scans are presented, though no quantitative data were derived for additional analysis from scanning electron microscopy (SEM) imagery.

### Leaf traits

After physiological measurements, all leaves were measured within 24 h for leaf area using a flatbed scanner to produce images. These images were then analysed for leaf area in the cuvette using ImageJ (Rueden *et al.* 2017), with values recorded. After measurement of area, leaves were kept in paper coin envelopes that were labelled and put in a drying oven for a minimum of 72 h at 60 °C prior to measurement of leaf dry mass. Once both area and mass were determined, we calculated the parameter LMA (g m^−2^).

### Statistical analysis

All data and statistical analysis were performed in R (version 4.0.4; R Core Team 2021). Prior to all further statistical analyses, variables were assessed for normality. All measured variables conformed to assumptions of normality; no transformations were applied. Experimental sites followed a randomized block design spread throughout the oak-dominated understory of Ordway, with paired replicates of species and age classes represented in each block. To analyse our results, we chose a mixed-model approach, as potential genetic and environmental similarities within each block could influence the variability of the data set; ‘experimental site’ was assigned as a random effect in the models. For physiological and leaf morphological variables, species (*P. serotina*, *R. cathartica*), age class (seedling, tree) and seasonal sampling period (Early and Mid season) were considered fixed effects for the mixed-model ANOVA, where interactions amongst fixed effects were also assessed. To compute the results of the mixed models, we used the R package *lme4*, and also the *car* package for computing significance of each fixed factor and their interaction (Bates *et al.* 2015; Fox and Weisberg 2019). *Post hoc* comparisons were made using Tukey’s test, and comparisons between only two factors, as with fluorescence values of leaves with and without galls, were compared with a *t*-test. We also calculated the percent change from Early to Mid season for photosynthetic variables as well, where the mean value for species at tree or seedling stage during the Early season measurement point was subtracted from the Mid season mean value, and this difference was divided by the Early season mean value. As percent changes worked only with mean values for each measurement combination, we could not quantify statistical results in these cases.

## Results

### Photosynthetic capacity in the understory

**Figure 1.**
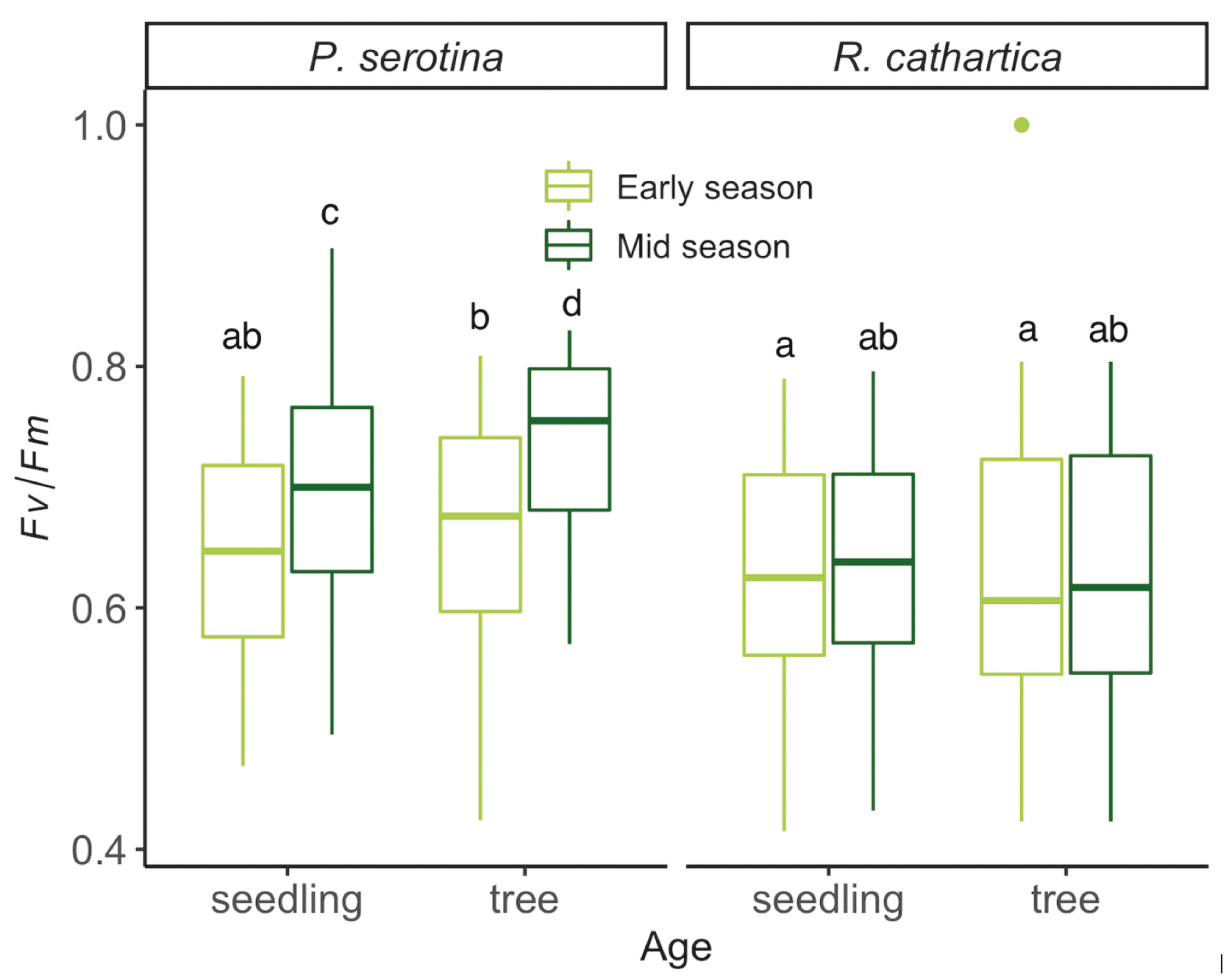
Boxplots indicating values of *F*_v_*/F*_m_ for leaves of both species for Early and Mid season timing of trees and seedlings. Statistical results from mixed-model ANOVA with species, age class, and seasonal timing as factors are reported in Table 1 and in the Results section. *Post hoc* analysis results are indicated using alphabetical notation where unshared letters amongst boxplots denote significant differences.

Our measurements of chlorophyll fluorescence in the leaves of both species at two seasonal time points in trees and seedlings found variation in *F*_v_/*F*_m_, a variable linked to photosynthetic capacity. Mixed-model ANOVA results show significant interaction effects of species and seasonal timing, as well as species and age class (Table 1; Fig. 1). Age class of the individuals sampled did not show the same strong trends over the entire data set (*P* = 0.07; Table 1), with *post hoc* tests amongst trees and seedlings for both measurement points and species showing *P* = 0.386. Our analysis also shows variation across the data set is driven more by *P. serotina*, while *F*_v_/*F*_m_ does not vary greatly across experimental factors in *R. cathartica* (Fig. 1). The significant interaction term is observed with increased *F*_v_/*F*_m_ in *P. serotina* across the growing season (Table 1; Fig. 1, *P* < 0.001), considering both age classes, that is not observed in *R. cathartica*. In *P. serotina*, there is a significant increase *F*_v_/*F*_m_ in from Early to Mid season (*P* < 0.001), whereas no shift in *F*_v_/*F*_m_ is detected in *R. cathartica* (Fig. 1).

**Table 1.** Summary of mixed-model ANOVA results for leaf variables detecting variation amongst both species at two measurement periods (seasonal timing = Early and Mid season) for trees and seedlings, where site was defined as a random effect. *P*-values (*P*) and chi-squared values (χ^2^) resulting from the analysis are reported, with statistical significance denoted with asterisks, where *P* < 0.05 (*), *P* < 0.01 (**) and *P* < 0.001 (***). For all factors of variation, degrees of freedom = 1.

	LMA		*V*_cmax_		*J*_max_		*F*_v_ */F*_m_		*A*_sat_		*R*_d_		CGE		*A*_400_		CGE_400_		Stomatal density	
	χ^2^	*P*	χ^2^	*P*	χ^2^	*P*	χ^2^	*P*	χ^2^	*P*	χ^2^	*P*	χ^2^	*P*	χ^2^	*P*	χ^2^	*P*	χ^2^	*P*
Species	118.98	***	1.26	0.26	4.05	*	110.35	***	1.12	0.29	0.44	0.51	0.03	0.85	1.32	0.25	0.01	0.92	10.47	**
Seasonal timing	64.92	***	1.09	0.29	1.24	0.26	73.79	***	5.22	*	1.78	0.18	1.17	0.27	4.04	*	0.90	0.34		
Age class	60.46	***	6.85	**	4.79	*	3.31	0.07	0.03	0.84	0.10	0.74	0.25	0.61	0.19	0.66	0.23	0.62	10.28	**
Species × seasonal timing	0.61	0.43	3.04	0.08	1.29	0.26	20.49	***	0.19	0.66	1.51	0.22	0.17	0.67	0.22	0.64	0.25	0.61		
Species × age class	21.61	***	0.04	0.84	1.39	0.24	13.17	***	0.90	0.34	0.79	0.37	0.46	0.49	1.67	0.19	0.34	0.55	1.26	0.26
Seasonal timing × age class	0.03	0.87	4.34	*	1.24	0.27	2.02	0.16	1.23	0.26	0.63	0.42	0.55	0.45	0.91	0.34	0.38	0.53		
Species × seasonal timing × age class	7.24	**	0.17	0.68	0.09	0.76	0.86	0.35	0.001	0.97	0.48	0.48	0.29	0.59	0.01	0.93	0.17	0.68		

**Figure 2.**
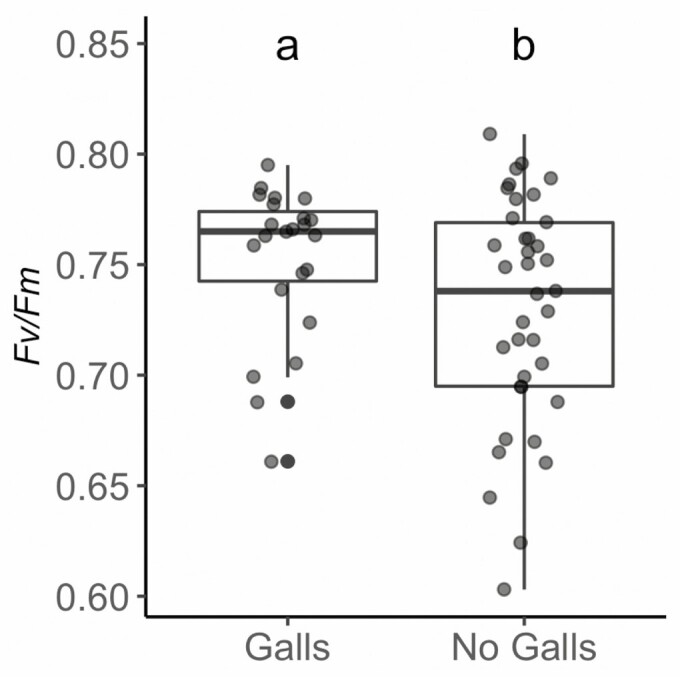
Dark-adapted *F*_v_*/F*_m_ for leaves sampled from individual trees of *P. serotina* sampled in the Early season. The influence of gall presence (‘Galls’) and absence (‘No Galls’) was assessed through a paired *t*-test to distinguish if this commonly occurring insect relationship alters measured rates of chlorophyll fluorescence; differences are indicated by unshared letters. Leaves infected with galls show a significantly higher rate of *F*_v_/*F*_m_ compared to leaves that were not infected (*P* < 0.05).

In May only (Early season), we measured *F*_v_/*F*_m_ in leaves of trees with gall formations on them (*n* = 60) to assess any potential variation that should be accounted for, even though all other physiological measurements (including *F*_v_/*F*_m_) reported in this study were only done on leaves with no galls present. Using a paired *t*-test to compare the presence and absence of galls (Fig. 2), we found a significant difference between the two groups in *F*_v_/*F*_m_ (*P* < 0.05, *t* = 2.031). Mean values of *F*_v_/*F*_m_ were actually higher in leaves of *P. serotina* that hosted galls, a surprising result.

Light response curves, which estimate the photosynthetic carbon assimilation and respiration rates under varying short-term experimental light conditions, yielded results that did not indicate significant species or age class differences, nor significant interactions amongst factors (Tables 1 and 2). We observed a significant difference in *A*_sat_ between Early and Mid season sampling periods considering both species and age classes, with higher rates of carbon uptake at mid-season (*P* < 0.05, *x*^2^ = 5.22, *d.f.* = 1). However, for all other variables estimated through our light curve analysis (*R*_d_, CGE, CGE_400_), we found no significant variation amongst inherent biological (species, age class) nor seasonal differences, nor significant interaction terms amongst experimental factors. Carbon assimilation at 400 μmol PAR (*A*_400_), which estimates activity under average understory light conditions, did exhibit a similar seasonal pattern to *A*_sat_: considering both trees and seedling of both species, Mid season values were greater than Early season values (*P* < 0.05; Tables 1 and 2). There were no significant interactions amongst variables for *A*_400_.

**Table 2.** Carbon cycling variables derived from photosynthetic light curves. Values presented are means with standard deviations for both species under all experimental conditions. For all variables except CGE and CGE_400_, which are ratios, the units are μmol CO_2_ m^−2^ s^−1^.

Species	Sampling period	Age class	*A*_sat_	*R*_d_	CGE	*A*_400_	CGE_400_
*P. serotina*	Early season	Seedling	7.82 ± 2.21	0.35 ± 0.18	0.96 ± 0.02	6.84 ± 1.69	0.95 ± 0.02
		Tree	8.47 ± 1.38	0.53 ± 0.5	0.97 ± 0.00	7.75 ± 0.94	0.97 ± 0.01
	Mid season	Seedling	8.35 ± 3.68	0.27 ± 0.18	0.97 ± 0.01	7.11 ± 2.72	0.97 ± 0.01
		Tree	9.93 ± 2.37	0.26 ± 0.06	0.97 ± 0.01	8.25 ± 1.54	0.97 ± 0.01
*R. cathartica*	Early season	Seedling	7.42 ± 0.82	0.34 ± 0.26	0.96 ± 0.03	6.56 ± 0.66	0.95 ± 0.04
		Tree	5.63 ± 1.03	0.29 ± 0.07	0.95 ± 0.02	5.14 ± 0.93	0.94 ± 0.02
	Mid season	Seedling	8.67 ± 1.75	0.32 ± 0.17	0.97 ± 0.01	7.55 ± 1.23	0.96 ± 0.02
		Tree	9.22 ± 3.8	0.35 ± 0.06	0.97 ± 0.03	6.71 ± 2.72	0.96 ± 0.03

**Figure 3.**
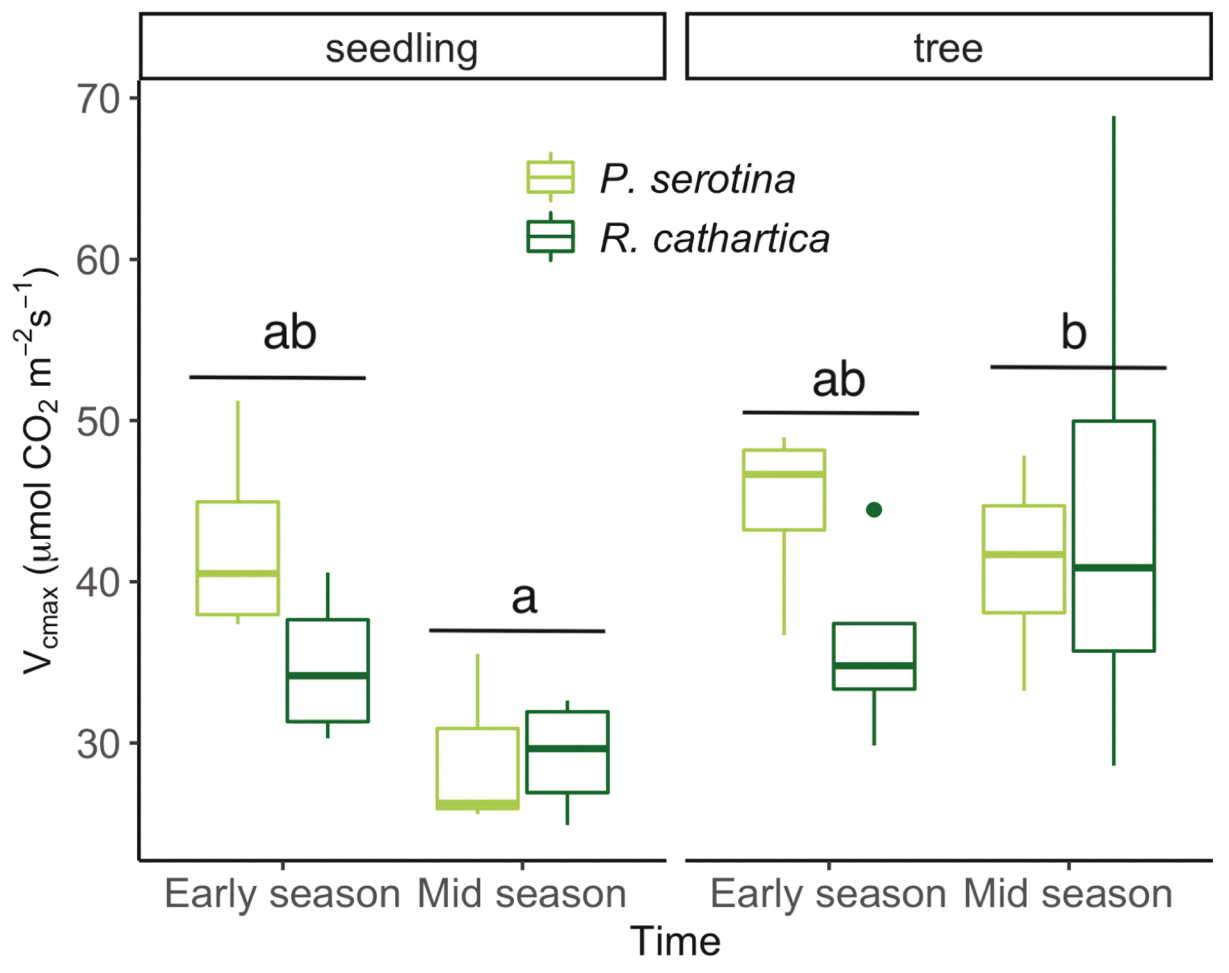
Values of maximum carboxylation (*V*_cmax_) rates in leaves of both species for Early and Mid season timing of trees and seedlings. Statistical results from *post hoc* Tukey’s HSD analysis are shown using alphabetical notation, where differences are indicated by unshared letters, where significance is defined at *P* < 0.05.

**Figure 4.**
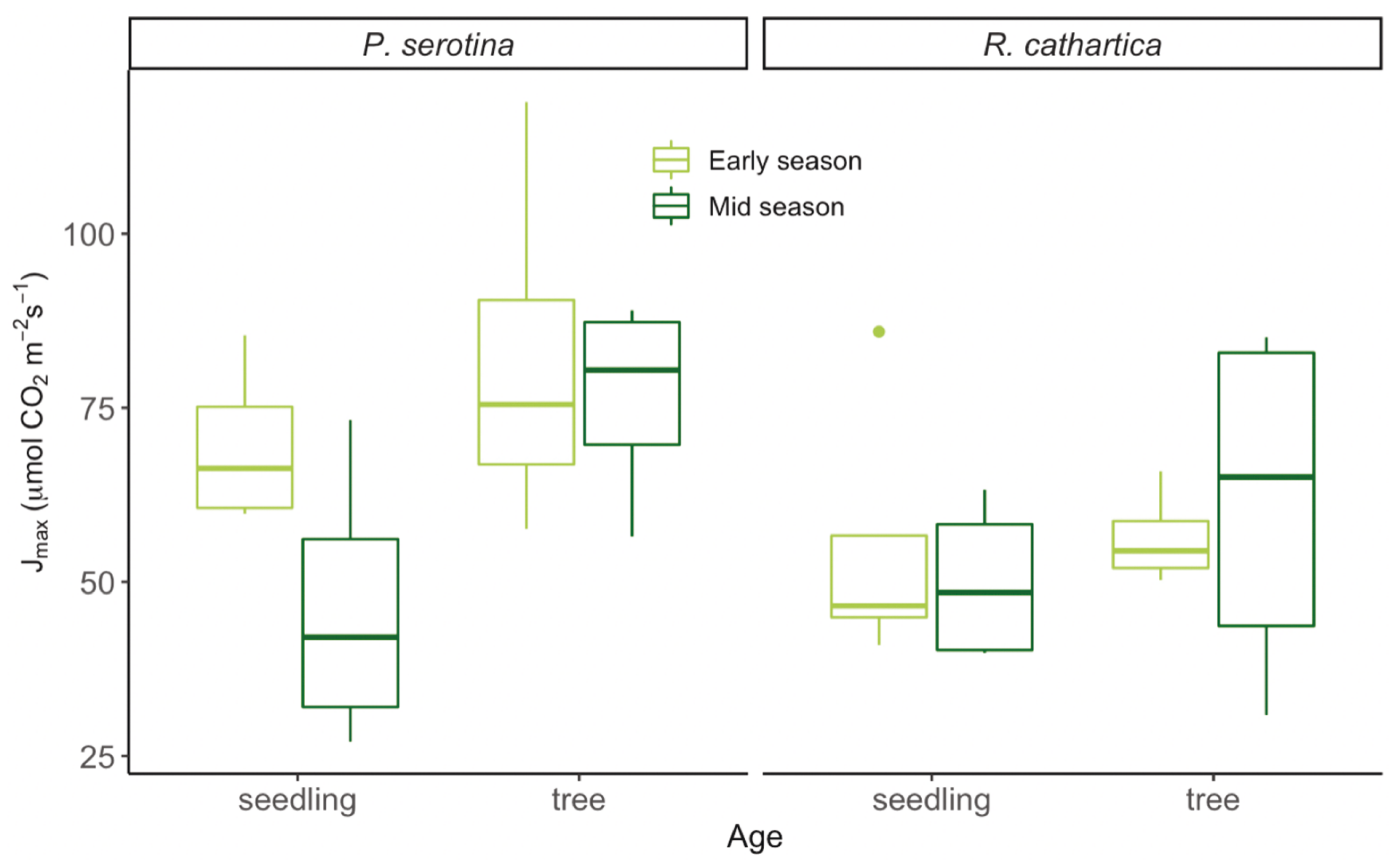
Maximum rate of electron transport (*J*_max_) for all experimental groups. Boxplots show mean values of data collected from both species for each measurement period for leaves of seedlings and trees. Mixed-model ANOVA and Tukey’s HSD analyses found only significant differences between age classes (seedling < tree; *P* < 0.05) and species, where *P. serotina* > *R. cathartica* (*P* < 0.05). No significant interaction terms amongst species, age class and seasonal timing were detected, and thus we did not include alphabetical notation on the plot to note the similarities amongst groups depicted.

We also examined photosynthetic capacity in terms of the response to short-term changes in CO_2_ to derive the variables *V*_cmax_ and *J*_max_ (Figs 3 and 4). Collected on the same leaves as the light response curves described above, the *V*_cmax_ data show a significant increase from seedlings to trees considering all data (Table 1; Fig. 3; *P* < 0.01, *x*^2^*=* 6.85). For *J*_max_, this increase across age classes was also observed considering all data (Table 1; Fig. 4, *P* < 0.05, *x*^2^ = 4.79). For *V*_cmax_, there was also a significant interaction between the seasonal timing and the age class of the sampled leaves (Table 1, *P* < 0.05, *x*^2^ = 4.34), driven primarily by the large differences between mid-season trees and seedlings, with trees showing much higher rates at that time period across both species (Fig. 3). Our Fig. 3 depicts all experimental combinations, which collectively did not have significant interactions, likely due to the lower sample size compared to our fluorescence measurements. There were no significant species differences for *V*_cmax_, though we did find higher rates of *J*_max_ in *P. serotina* (Fig. 4; Table 1) considering all data. No significant interaction effects were observed for the variable *J*_max_ (Fig. 4).

**Figure 5.**
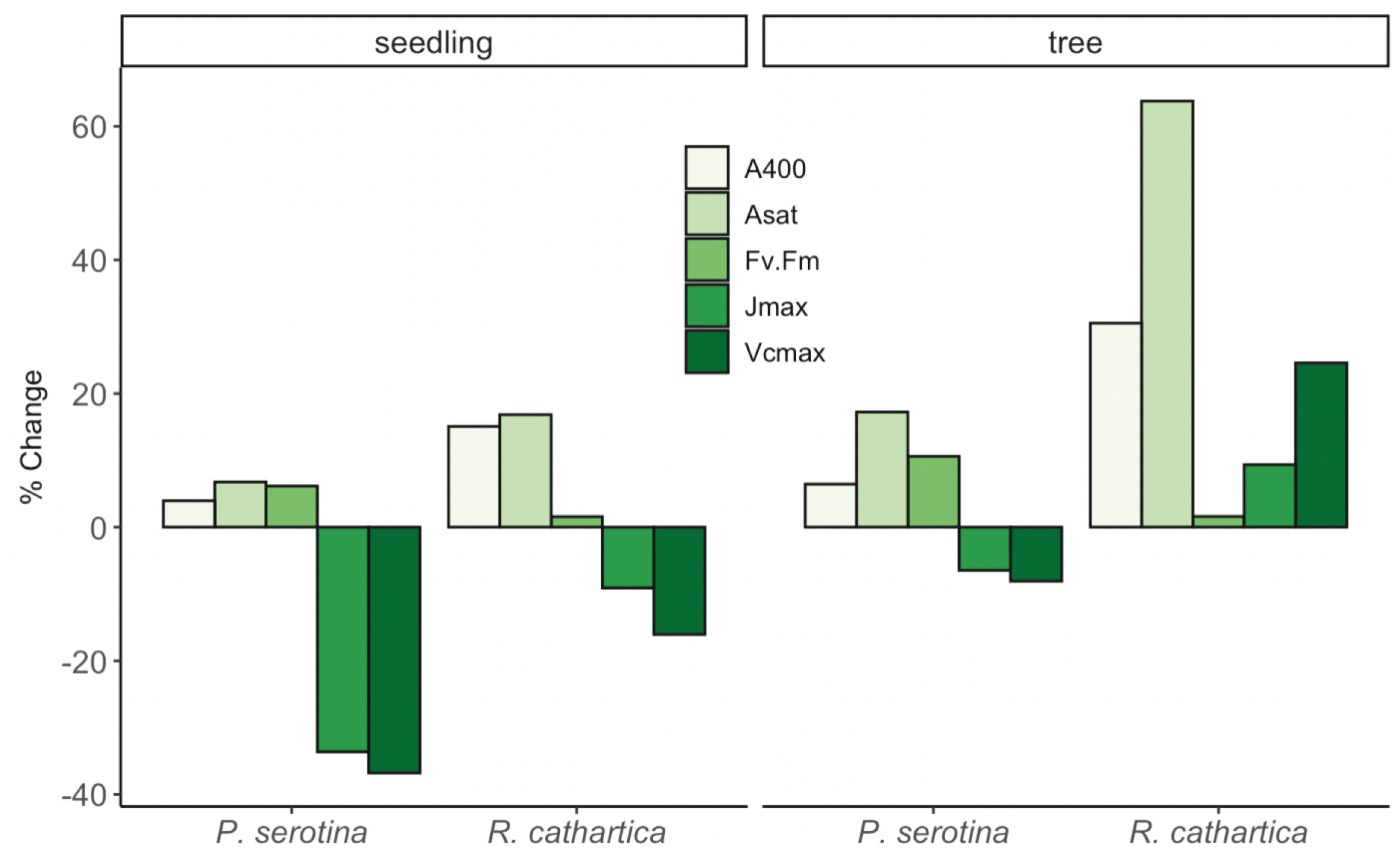
Percent change in mean values of photosynthetic variables across the growing season. Values shown are percent change between Early and Mid season of the mean values for both species of both age classes. A positive percent change represents an increase in the variables’ value from Early to Mid season, and a negative percent change indicates a decrease in the variables’ value.

Our study also examined trends of photosynthesis across variables through the season to increase understanding in their relation to each other. Figure 5 presents the percent change in the mean values for each species and age class from Early to Mid season. The greatest relative changes are observed in the increases in light response curve parameters *A*_sat_ and *A*_400_ in leaves from *R. cathartica* trees, and a much-reduced version of this trend is observed in *R. cathartica* seedlings as well as the leaves from seedlings and trees of *P. serotina* (Fig. 5). Another distinctive trend is found in the declining response of *V*_cmax_ and *J*_max_ across the growing season in all leaves except those of *R. cathartica* trees, which increase their values nearly 10 and 20 %, respectively. In fact, only in leaves of *R. cathartica* trees do all photosynthetic variables increase through the growing season, with other species–age combinations showing mixed responses across variables (Fig. 5).

### Leaf morphological traits

**Figure 6.**
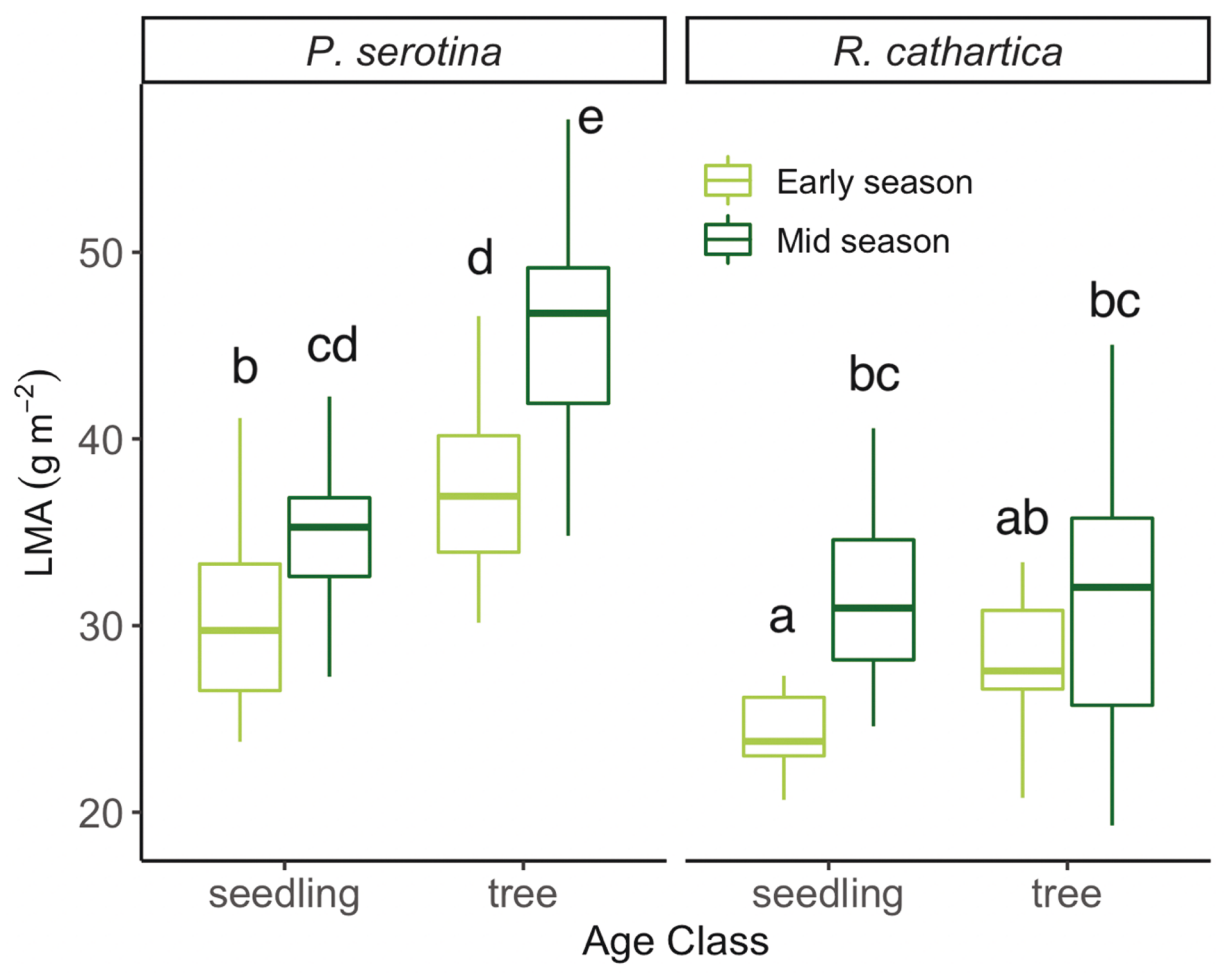
Leaf mass per area (LMA) for both species for Early and Mid season time points at both age classes. Results of *post hoc* analysis are denoted with alphabetical notation, where unshared letters indicate significant differences, as defined by *P <* 0.05.

Leaf mass per area, a measure of leaf thickness, which relates closely to development and environmental influences in deciduous species, varied significantly amongst the two species, age classes, and seasonal timing in our study (Table 1; Fig. 6). However, how LMA varied by age class within species differed, with *P. serotina* exhibiting much greater differences between seedlings and trees (Fig. 6) than in leaves of *R. cathartica*. Largest seasonal differences were observed in leaves from trees of *P. serotina* (*P* < 0.0001) and seedlings of *R. cathartica* (*P* < 0.001), whereas seedlings of *P. serotina* and trees of *R. cathartica* showed no significant variation in LMA across the season (both *P* > 0.05). Trees (but not seedlings) of *P. serotina* significantly increased their LMA through the season, as did seedlings (but not trees) of *R. cathartica* (Fig. 6; Table 1). Across all experimental groups, leaves increased LMA from May to June measurement points (*P* < 0.0001), and *P. serotina* had higher values of LMA (*P* < 0.0001) considering all measurements.

**Figure 7.**
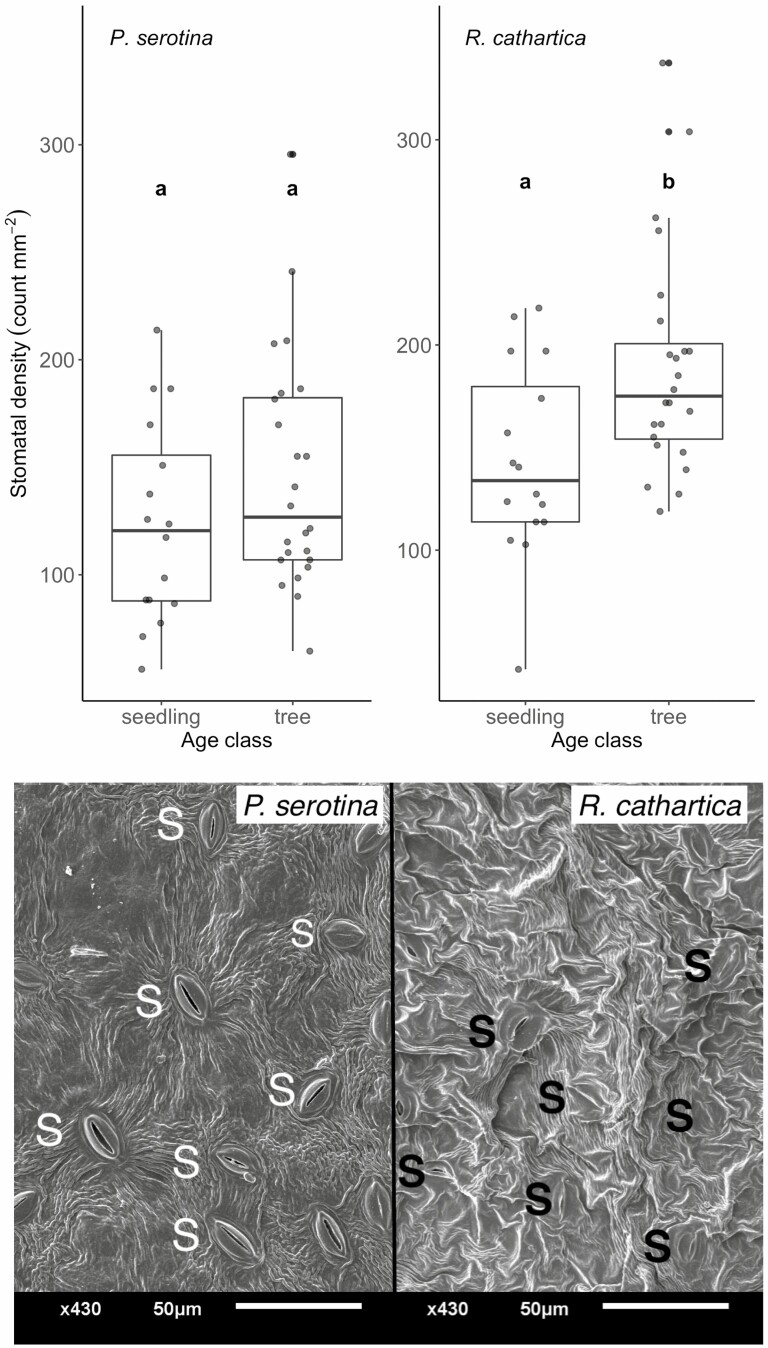
Stomatal density in leaves of seedlings and trees of *P. serotina* and *R. cathartica.* The upper panel shows boxplots show the mean (midline), and first (lower) and third (upper) quartiles of the data; alphabetical notation indicates significance amongst species and age class at *P* < 0.05 (*n* = 40 for each species). The lower panel shows images of leaf stomata of both species, sampled Mid season. Abaxial sides of leaves from trees of both species imaged via SEM under ×430 magnification. Stomata are identified with an ‘S’ (white on *P. serotina*, black on *R. cathartica*). These images were not used for stomatal density analyses, but to qualitatively visualize the different morphology of stomata in the species.

Our study also probed the potential variation in stomata in both species, though only at a single measurement point (Fig. 7). Stomatal density varied amongst different factors: considering all measurements (*n* = 80), trees had higher density than seedlings (*P* < 0.01, *x*^2^ = 10.28, *d.f.* = 1) and *R. cathartica* had higher density than *P. serotina* (*P* < 0.01, *x*^2^ = 10.47, *d.f.* = 1), and no significant interaction term amongst factors was detected. We found significant differences between leaves of seedlings and trees of *R. cathartica*, where trees had significantly higher stomatal density than all other sampled species–age class combinations (*P* < 0.05). We did not find differences amongst leaves of seedlings and trees of *P. serotina* and *R. cathartica* seedlings (Fig. 7). When visualizing leaves of trees using SEM (Fig. 7), we noted qualitative differences in the stomatal morphology of each species, though did not use these images for further quantification.

## Discussion

Capturing the shifting ecological and carbon cycling baselines of a temperate deciduous forest understory with a large non-native invasive species presence remains a challenge (Godefroid *et al.* 2005; Xu *et al.* 2007b; Heberling *et al.* 2019). Our field-based study addresses multiple axes of variation that contribute to the strategies of commonly occurring understory species in terms of their leaf-level physiology, which can be the foundation for how they assimilate and allocate carbon at the organismal scale. Specifically, we examined how two species—one native (*P. serotina*) and one introduced and invasive (*R. cathartica*), perform photosynthesis at two distinct developmental time points (seedling and subcanopy tree/shrub), during the Early and Mid growing season, and used the setting of the forest, not controlled plots, to directly measure and estimate parameters of the light and carbon reactions of photosynthesis. The benefit of measuring multiple photosynthetic parameters collectively in a field-grown, understory forest site at multiple measurement conditions is the authenticity of physiological data. However, this approach can present an entanglement of environmental and biological drivers, within and across estimates of the photosynthetic light and carbon reactions, which may be more challenging to parse than studies that employ common garden experiments, such as Harrington *et al.* (1989) and Heberling *et al.* (2019). Our results, controlled by our block design, suggest complex, varied influences on photosynthesis that indicate environmental and biological plasticity which may contribute to the historic and continued expansion of *R. cathartica* in the US Upper Midwest region.

### Variability in photosynthetic responses during the understory growing season

The design of our study sought to compare two species that represent differences in endemicity, seasonal and developmental time points, and age classes (therefore structural and developmental differences), with the working hypotheses that the common invasive *R. cathartica* would employ a more ruderal-style growth strategy, discernible through its carbon gain strategies (Grime 1977; Grubb *et al.* 1996; Knight *et al.* 2007; van Kleunen *et al.* 2010). Further, evidence from previous studies suggested that invasive understory plants would exhibit more adaptive shift in photosynthetic rates from the start of the growing season (open canopy) to middle season (closed canopy) compared to the native species, maximizing carbon assimilation when light is most available and also extending its leaf-out phenology earlier into the spring (Harrington *et al.* 1989; Xu *et al.* 2007a; van Kleunen *et al.* 2010; Heberling *et al.* 2019). Interestingly, *P. serotina* (endemic to North America) can assume these adaptive growth and shade tolerance traits when it grows as a non-native invasive in Europe, suggesting a plasticity that is also mediated by local environment and plant communities (Abrams *et al.* 1992; Godefroid *et al.* 2005; Closset-Kopp *et al.* 2011).

Our results do not completely depart from the established paradigms, but do add more specific nuance to understanding how understory species shift their metabolism across the season. We found fluorescence rates to be higher in the native *P. serotina*, as well as more seemingly flexible—they increased significantly from Early to Mid season, whereas fluorescence of *R. cathartica* remained at a similar rate across seasonal sampling points. We anticipated the invasive *R. cathartica* to either have higher rates earlier in the season to take advantage of understory light availability pre-canopy closure (Heberling *et al.* 2019) or alternatively to increase rates as the season progressed due to leaf development. While the observed consistency in fluorescence in *R. cathartica* from Early to Mid season did not support these hypotheses, other photosynthetic variables adjusted rates significantly with seasonal progress. In both species, *A*_sat_ and *A*_400_ increased through the growing season across both species and age classes. This seasonal shift towards higher rates in photosynthesis is not uncommon for species that experience increasing light exposure through the growing season, and may be maximized to align with the greatest values of solar irradiance around the solstice (Bauerle *et al.* 2012; Way *et al.* 2017). The largest percentage increase in *A*_sat_ and *A*_400_ was observed in leaves from trees of *R. cathartica*, nearly doubling for *A*_sat_. So despite its earlier leaf-out phenology, our data suggest leaves of *R. cathartica* trees may still optimize carbon assimilation rates at the middle of the growing season once PAR and air temperatures are highest, showing productivity under multiple ecological scenarios in the understory (Knight *et al.* 2007). We do note a lack of variation in respiration rates and CGE at saturating and moderate PAR levels. As CGE relies on carbon assimilated and carbon released via respiration, the stability across the season suggests decoupled trends in these aspects of carbon cycling that result in a maintained rate, which has been observed in other temperate species across the growing season (Heskel and Tang 2018).

Adding further complexity, we found no significant seasonal trends in *V*_cmax_ and *J*_max_ across both species and age class, though an overall significant difference between trees and seedlings for both variables. Of all the treatment groups, only *R. cathartica* trees increased the rates of *V*_cmax_ and *J*_max_ across the growing season (shown as percent change in Fig. 5). Paired with our *A*_sat_ data (though curiously, not fluorescence), this supports the idea of non-native understory plants being more plastic under the variable light environment across the growing season compared to native plants (Martinez and Fridley 2018; Heberling *et al.* 2019). When we parse the differences in these variables across seedlings and trees, it suggests the influential role of the light environment on driving metabolic and leaf traits in these species, regardless of native/non-native status (Augspurger and Bartlett 2003; Sefcik *et al.* 2006; De Pauw *et al.* 2022). Leaves of Early season seedlings of *P. serotina* have rates of carboxylation similar to those of *P. serotina* trees at Early and Mid season, though there is a significant reduction in seedling rates from Early to Mid season (a similar trend observed for *R. cathartica*). Prior to full canopy leaf-out, it is likely the light extinction from mid- to lower canopy levels (i.e. what understory trees and seedlings experience, respectively) may be more similar than later in the season (Canham 1988; Wei *et al.* 2017), after full leaf-out, though only leaves of *R. cathartica* trees maintain or even slightly enhance rates during this transition. It may be that more than just the stature (therefore light access), of being a tree matters, but also other traits that may be enhanced in a larger, subcanopy species (Augspurger and Bartlett 2003; Falster *et al.* 2018), including nutrient and water acquisition through more expansive root networks, reduced intra-species competition, and reduced herbivory, which are generally exhibited by the non-native *R. cathartica* (Knight *et al.* 2007).

### Linking leaf traits to growth in the understory

Our study also assessed certain physical leaf traits that are likely to mediate how light is captured (galls in *P. serotina*) (Larson 1998), how the rate of CO_2_ assimilation into the mesophyll (stomatal density) (Sakoda *et al.* 2020), and one of the most common traits used in modelling and scaling (LMA) (Wright *et al.* 2004; Keenan and Niinemets 2016). It is important to note how common the presence of galls is on *P. serotina* leaves at this site and in similar forests in this region. As our goal was to characterize and assess the understory carbon cycling in these species, it would feel like an oversight to only measure leaves of *P. serotina* without galls. We assumed the presence of galls in *P. serotina* would cause a reallocation of photosynthetic resources and decrease fluorescence rates compared to leaves without galls (note: we only used non-galled leaves to compare rates across age classes, species and sampling periods, though galls were very common, especially in trees). Surprisingly, we found galled leaves actually had significantly higher rates of *F*_v_/*F*_m_ than non-galled leaves. A study focusing on these differences in *P. serotina* hypothesized a source–sink dynamic that would enhance photosynthesis in ungalled leaves on the same shoot as galled leaves, though they also found a higher rate of photosynthesis in leaves of shoots that had no gall formation at all (Larson 1998). While galls may decrease rates of photosynthesis by altering both the leaf architecture and resource allocation (Larson 1998; Kmieć *et al.* 2018), there is also evidence that these gall-forming insects may have a more mutualistic relationship with plants, exchanging nutrients and potentially enhancing photosynthesis (Chen *et al.* 2020; Murakami *et al.* 2021). The latter relationship may be suggested by our fluorescence results, and is worthy of more examination in *P. serotina*.

Our examination of stomatal density in the leaves from seedlings and trees from both species found a close connection to trends observed in parameters derived from light and carbon response curves. Specifically, we found significantly higher stomatal density in leaves from trees of *R. cathartica* compared to seedlings of the same species, and compared to trees and seedlings of *P. serotina*, which aligns with the greater increases rates of *A*_sat_, *A*_400_, *Vc*_max_ and *J*_max_ observed. These responses suggest a morphological coupling that *R. cathartica* may employ to increase CO_2_ conductance once *R. cathartica* establishes a mid-canopy stature. As light becomes less limiting in the mid-canopy, there is a shift to optimize stomatal conductance and carbon fixation (Gay and Hurd 1975; Wong *et al.* 1979). That we do not observe this same shift in *P. serotina* under similar mid-canopy conditions is curious, but may imply a reduced trait plasticity in the native species compared to the non-native, especially when that trait relates closely to carbon gain and growth (Xu *et al.* 2007a; van Kleunen *et al.* 2010; Martinez and Fridley 2018). Leaf mass per area (and by extension, its inverse specific leaf area) was likely the most variable trait we measured amongst all experimental factors: broadly, leaves showed greater values, and therefore thicker leaves, in trees, in Mid season and in *P. serotina*. The seasonal increase, as well as that observed for trees, aligns with understanding on leaf development through the season and response to increased sunlight—leaves generally have higher LMA values under sun compared to shade due to longer and/or more layers of palisade cells (Niinemets *et al.* 1998; Poorter *et al.* 2009). The range in values observed in these two species measured only 6 weeks apart in trees and seedlings emphasizes the need to quantify and incorporate the variation observed in shaded and understory species into ecosystem models to more accurately capture how this trait varies in its relation to photosynthesis and growth (Niinemets *et al.* 2015; Keenan and Niinemets 2016).

Trends in community cover shifts observed at our study site and more broadly in the Upper US Midwest indicate the establishment and expansion of the invasive shrub *R. cathartica* as well as other common understory invasives, altering the historic forest composition (Knight *et al.* 2007; Anderson *et al.* 2019). While assessing multiple parameters that characterize photosynthetic capacity allows us to better understand the carbon cycling of these species, these estimates require additional ecological, environmental and biological information to relate to the observed growth and shifts at the organismal and community scales (Grubb *et al.* 1996; Godefroid *et al.* 2005; Whitfeld *et al.* 2014; Martinez and Fridley 2018; De Pauw *et al.* 2022). Individual carbon allocation and growth strategies may allow for certain species to accelerate across life history stages (i.e. seedling to tree), and this may also be mediated by external pressures such as herbivory, extreme or altered weather, or enhanced light accessibility of a new forest gap (Grime 1977; Knight *et al.* 2007). Though we may lack the data in this study to relate our findings from one season to the entire growth strategies of these species across a longer time scale, our data do support connections between leaf-level carbon cycling physiology, the fundamental metabolism of growth, to organismal and community trends observed in these native and non-native understory species. Understanding how these and other understory species use and allocate carbon and resources across the growing season will provide information needed for modelling the community and organismal transitions between seedling growth at the understory and establishment as mid-canopy trees. Specifically, ecosystem demography models and ‘two-leaf’, multi-layer canopy models can use this and similar data to inform both plant functional type and trait values that expand beyond deciduous trees in the overstory. As temperate forests continue to shift due to both increasing composition of non-native species and altered climatic conditions, it is crucial to monitor, model and forecast how they cycle and store carbon.

## Data Availability

Data are collected for this manuscript are openly downloadable and citable via Zenodo (doi: 10.5281/zenodo.6803562).

## Notes

**Form & Function. Chief Editor:** Kate McCulloh
